# Supplementary material for: Medicines prescribed for asthma, discontinuation and perinatal outcomes, including breastfeeding: A population cohort analysis
Source: PLoS One. 2020 Dec 9;15(12):e0242489. doi: 10.1371/journal.pone.0242489 (PMC7725302; doi:10.1371/journal.pone.0242489)
Supplement: S1 Table — (DOCX) [file pone.0242489.s002.docx]

#### S1 Table Demographic details of population, excluding congenital anomalies [n = 113316]

|  | **Population [excluding TOPFAs and those with congenital anomalies]** | **Any asthma medicine [R03 PRE4-T3]** | **Unmedicated asthma [R03 PRE4-1 and not T123]** | **Any asthma medicine t1-t3 [R03]** | **SABA t1-t3** | **Only SABA prescribed** | **LABA t1-t3**  **[R03AC12,13]** | **ICS t1-t3 [R03BA]** | **Only ICS prescribed** | **LKA t1-t3 [R03DC]** | **OCS t1-t3 + another asthma medicine**  **[H02AB + R03]** |
| --- | --- | --- | --- | --- | --- | --- | --- | --- | --- | --- | --- |
| **Number of infants** | n[%] | n[%] | n[%] | n[%] | n[%] | n[%] | n[%] | n[%] | n[%] | n[%] | n[%] |
| **Total** | 113316 [100] | 13516 [100] | 3850 [100] | 9666 [100] | 8942 [100] | 4044 [100] | 493 [100] | 4132 [100] | 393 [100] | 101 [100] | 552 [100] |
| Singletons | 110,727 [97.72] | 13221 [97.82] | 3748 [97.35] | 9473 [98.00] | 8765 [98.02] | 3973 [98.24] | 483 [97.97] | 4032 [97.58] | 383 [97.46] | 97-100 [<100] | 544 [98.55] |
| Twins and higher order multiples [number of infants] | 2589 [2.28] | 295 [2.18] | 102 [2.65] | 193 [1.99] | 177 [1.98] | 71 [1.76] | 10 [2.03] | 100 [2.42] | 10 [2.54] | <5 [<5] | 8 [1.45] |
| **Infant outcome** |  |  |  |  |  |  |  |  |  |  |  |
| Live birth | 112,885 [99.62] | 1,3441 [99.45] | 3823 [99.30] | 9618 [99.50] | 8897 [99.50] | 4026 [99.55] | 493 [100] | 4112 [99.52] | 393 [100] | 101 [100] | 545 [98.73] |
| Stillborn | 431 [0.38] | 75 [0.55] | 27 [0.70] | 48 [0.50] | 45 [0.50] | 18 [0.45] | 0 [0] | 20 [0.48] | 0 [0] | 0 [0] | 7 [1.27] |
| **Exposures** |  |  |  |  |  |  |  |  |  |  |  |
| Number of infants whose mothers' records included **heavy drinking and/or substances misuse** | 1795 [1.58] | 304 [2.25] | 84 [2.18] | 220 [2.28] | 209 [2.34] | 91 [2.25] | 14 [2.84] | 98 [2.37] | 6 [1.53] | <5 [<5] | 11 [1.99] |
| Number of infants exposed to **insulin** in **pre1 and tri1** | 367 [0.32] | 56 [0.41] | 15-18 [<1] | 41-44 [<0.5] | 38-41 [<0.5] | 8-11 [<0.5] | <5 [<1] | 16-19 [<0.5] | <5 [<1] | <5 [<5] | 5 [0.91] |
| Number of infants exposed to **AEDs** in **pre1 and tri1** | 659 [0.58] | 118 [0.87] | 39 [1.01] | 79 [0.82] | 74 [0.83] | 38 [0.94] | <5 [<1] | 30 [0.73] | <5 [<1] | <5 [<5] | 6 [1.09] |
| Number of infants exposed to **coumarins** in **pre1 and tri1** | 48 [0.04] | 8 [0.06] | <5 [<1] | <5 [<0.1] | <5 [<0.1] | <5 [<0.2] | 0 [0] | <5 [<0.2] | 0 [0] | <5 [<5] | <5 [<0.80] |
| **Parity:** |  |  |  |  |  |  |  |  |  |  |  |
| Primiparous | 48,332 [42.65] | 5769 [42.68] | 1741 [45.22] | 4028 [41.67] | 3732 [41.74] | 1699 [42.01] | 190 [38.54] | 1758 [42.55] | 168 [42.75] | 37 [36.63] | 173 [31.34] |
| Multiparous | 64,984 [57.35] | 7747 [57.32] | 2109 [54.78] | 5638 [58.33] | 5210 [58.26] | 2345 [57.99] | 303 [61.46] | 2374 [57.45] | 225 [57.25] | 64 [63.37] | 379 [68.66] |
|  |  |  |  |  |  |  |  |  |  |  |  |
| **Mean age** at pregnancy end [years: [SD]] | 28.22 [6.06] | 27.71 [6.13] | 27.20 [6.12] | 27.92 [6.13] | 27.84 [6.11] | 27.82 [6.10] | 28.48 [6.09] | 27.79 [6.18] | 28.29 [6.16] | 29.03 [5.94] | 28.68 [6.35] |
| Maternal age at pregnancy end [years] |  |  |  |  |  |  |  |  |  |  |  |
| <20 | 9552 [8.43] | 1342 [9.93] | 434 [11.27] | 908 [9.39] | 848 [9.48] | 377 [9.32] | 30 [6.09] | 419 [10.14] | 38 [9.67] | 6 [5.94] | 46 [8.33] |
| 20-24 | 23,967 [21.15] | 3118 [23.07] | 985 [25.58] | 2133 [22.07] | 2006 [22.43] | 918 [22.70] | 107 [21.70] | 931 [22.53] | 77 [19.59] | 16 [15.84] | 103 [18.66] |
| 25-29 | 308,93 [27.26] | 3760 [27.82] | 1057 [27.45] | 2703 [27.96] | 2509 [28.06] | 1154 [28.54] | 156 [31.64] | 1120 [27.11] | 111 [28.24] | 31 [30.69] | 150 [27.17] |
| 30-34 | 30,332 [26.77] | 3259 [24.11] | 857 [22.26] | 2402 [24.85] | 2199 [24.59] | 976 [24.13] | 108 [21.91] | 1014 [24.54] | 102 [25.95] | 27 [26.73] | 142 [25.72] |
| 35-39 | 15,437 [13.62] | 1686 [12.47] | 434 [11.27] | 1252 [12.95] | 1142 [12.77] | 512 [12.66] | 70 [14.20] | 541 [13.09] | 55 [13.99] | 15 [14.85] | 87 [15.76] |
| 40-44 | 2986 [2.64] | 332 [2.46] | 79-82 [<5] | 251 [2.60] | 222 [2.48] | 100 [2.47] | 18-21 [<5] | 102 [2.47] | 10 [2.54] | 6 [5.94] | 19-22 [2.60] |
| >44 | 141 [0.12] | 15-18 [<1] | <5 [<1] | 13-16 [<0.5] | 12-15 [<0.5] | 7 [0.17] | <5 [<1] | <5 [<0.5] | 0 [0] | 0 [0] | <5 |
| unknown | 8 [0.01] | <5 [<1] | 0 [0] | <5 [<0.2] | <5 [<0.2] | 44 [1.09] | 0 [0] | <5 [<0.5] | 0 [0] | 0 [0] | 0 |
| **Smoking status** |  |  |  |  |  |  |  |  |  |  |  |
| non-smoker | 63,442 [55.99] | 6964 [51.52] | 1959 [50.88] | 5005 [51.78] | 4580 [51.22] | 2030 [50.20] | 263 [53.35] | 2139 [51.77] | 241 [61.32] | 64 [63.37] | 259 [46.92] |
| current smoker | 33,529 [29.59] | 4575 [33.85] | 1342 [34.86] | 3233 [33.45] | 3045 [34.05] | 1423 [35.19] | 149 [30.22] | 1394 [33.74] | 103 [26.21] | 23 [22.77] | 205 [37.14] |
| ex-smoker | 14,074 [12.42] | 1861 [13.77] | 511 [13.27] | 1350 [13.97] | 1247 [13.95] | 547 [13.53] | 76 [15.42] | 575 [13.92] | 45-48 [<15] | 10-13 [<15] | 84-87 |
| unknown | 2271 [2.00] | 116 [0.86] | 38 [0.99] | 78 [0.81] | 70 [0.78] | 44 [1.09] | 5 [1.01] | 24 [0.58] | <5 [<2] | <5 [<5] | <5 |
| **Socioeconomic status** |  |  |  |  |  |  |  |  |  |  |  |
| Wales Townsend fifth = 1 [least deprived] | 19,728 [17.41] | 2108 [15.60] | 587 [15.25] | 1521 [15.74] | 1373 [15.35] | 655 [16.20] | 80 [16.23] | 650 [15.73] | 84 [21.37] | 13 [12.87] | 70-73 |
| 2 | 21,649 [19.10] | 2375 [17.57] | 738 [19.17] | 1637 [16.94] | 1504 [16.82] | 708 [17.51] | 82 [16.63] | 660 [15.97] | 66-69 [<20] | 15 [14.85] | 94 [17.03] |
| 3 | 22,081 [19.49] | 2657 [19.66] | 783 [20.34] | 1874 [19.39] | 1734 [19.39] | 812 [20.08] | 76-79 [<20] | 759 [18.37] | 71 [18.07] | 19 [18.81] | 103 [18.66] |
| 4 | 24,226 [21.38] | 2978 [22.03] | 793 [20.60] | 2185 [22.61] | 2024 [22.63] | 874 [21.61] | 109 [22.11] | 948 [22.94] | 88 [22.39] | 29 [28.71] | 135 [24.46] |
| 5 [most deprived] | 25,179 [22.22] | 3346 [24.76] | 924 [24] | 2422 [25.06] | 2282 [25.52] | 984 [24.33] | 142 [28.80] | 1100 [26.62] | 80 [20.36] | 25 [24.75] | 146 [26.45] |
| Unknown | 453 [0.40] | 52 [0.38] | 25 [0.65] | 27 [0.28] | 25 [0.28] | 11 [0.27] | <5 [<5] | 15 [0.36] | <5 [<2] | 0 [0] | <5 |
| Townsend score, mean [SD] | 0.30 [3.17] | 0.55 [3.19] | 0.49 [3.20] | 0.57 [3.19] | 0.62 [3.20] | 0.51 [3.19] | 0.82 [3.39] | 0.68 [3.23] | 0.07 [3.02] | 0.57 [2.72] | 0.77 [3.19] |
| Townsend rank for Wales, mean [SD] | 3.12 [1.41] | 3.23 [1.40] | 3.19 [1.39] | 3.24 [1.41] | 3.26 [1.40] | 3.20 [1.41] | 3.31 [1.45] | 3.29 [1.42] | 3.03 [1.44] | 3.38 [1.35] | 3.34 [1.37] |
| **Mean time on database at LMP [years]** | 7.63 [4.94] | 8.09 [5.05] | 8.08 [4.98] | 8.10 [5.08] | 8.14 [5.10] | 7.91 [4.97] | 7.40 [4.94] | 7.98 [5.01] | 7.05 [4.53] | 9.25 [5.74] | 8.0. [5.24] |
| **Pregnancy end date in** |  |  |  |  |  |  |  |  |  |  |  |
| 2000 | 8024 [7.08] | 777 [5.75] | 206 [5.35] | 571 [5.91] | 511 [5.715] | 280 [6.92] | 31 [6.29] | 268 [6.49] | 45 [11.45] | <5 [<5] | 26 [4.71] |
| 2001 | 8327 [7.35] | 861 [6.37] | 238 [6.18] | 623 [6.45] | 568 [6.35] | 275 [6.80] | 48 [9.74] | 315 [7.62] | 38 [9.67] | <5 [<5] | 20 [3.62] |
| 2002 | 8620 [7.61] | 898 [6.64] | 255 [6.62] | 643 [6.65] | 581 [6.50] | 272 [6.73] | 41 [8.32] | 333 [8.06] | 43 [10.94] | <5 [<5] | 25 [4.53] |
| 2003 | 9568 [8.44] | 1109 [8.21] | 297 [7.71] | 812 [8.40] | 751 [8.40] | 345 [8.53] | 64 [12.98] | 393 [9.51] | 37 [9.41] | <5 [<5] | 32 [5.80] |
| 2004 | 10,300 [9.09] | 1239 [9.17] | 355 [9.22] | 884 [9.15] | 813 [9.09] | 363 [8.98] | 56 [11.36] | 420 [10.16] | 36 [9.16] | 7 [6.93] | 50 [9.06] |
| 2005 | 10,647 [9.40] | 1273 [9.42] | 331 [8.60] | 942 [9.75] | 886 [9.91] | 405 [10.01] | 48 [9.74] | 415 [10.04] | 33 [8.40] | <5 [<5] | 46 [8.33] |
| 2006 | 11,047 [9.75] | 1403 [10.38] | 404 [10.49] | 999 [10.34] | 916 [10.24] | 414 [10.24] | 61 [12.37] | 419 [10.14] | 44 [11.20] | 13 [12.87] | 63 [11.41] |
| 2007 | 11,511 [10.16] | 1465 [10.84] | 428 [11.12] | 1037 [10.73] | 965 [10.79] | 430 [10.63] | 44 [8.92] | 413 [1.00] | 32 [8.14] | 6 [5.94] | 66 [11.96] |
| 2008 | 11,697 [10.32] | 1483 [10.97] | 466 [12.10] | 1017 [10.52] | 957 [10.70] | 403 [9.97] | 35 [7.10] | 384 [9.29] | 24 [6.11] | 22 [21.78] | 63 [11.41] |
| 2009 | 11,562 [10.20] | 1411 [10.44] | 396 [10.29] | 1015 [10.50] | 954 [10.67] | 421 [10.41] | 31 [6.29] | 349 [8.45] | 25 [6.36] | 17 [16.83] | 75 [11.96] |
| 2010 | 12,013 [10.60] | 1597 [11.82] | 474 [12.31] | 1123 [11.62] | 1040 [11.63] | 436 [10.78] | 34 [6.90] | 423 [10.24] | 36 [9.16] | 22 [21.78] | 86 [15.58] |
| **Body mass index [BMI] nearest before LMP** |  |  |  |  |  |  |  |  |  |  |  |
| <20 | 9221 [8.14] | 970 [7.18] | 316 [8.21] | 654 [6.77] | 598 [6.69] | 257 [6.36] | 33 [6.70] | 296 [7.16] | 32 [8.14] | 10 [9.90] | 42 [7.61] |
| 20 – 24 | 33,579 [29.63] | 3892 [28.80] | 1132 [29.40] | 2760 [28.55] | 2525 [28.24] | 1159 [28.66] | 125 [25.35] | 1157 [28.00] | 128 [32.57] | 20 [19.80] | 131 [23.73] |
| 25 – 29 | 17,863 [15.76] | 2468 [18.26] | 689 [17.90] | 1779 [18.40] | 1662 [18.59] | 717 [17.73] | 97 [19.68] | 762 [18.44] | 67 [17.05] | 23 [22.77] | 106 [19.20] |
| 30 – 34 | 7588 [6.70] | 1224 [9.06] | 320 [8.31] | 904 [9.35] | 838 [9.37] | 345 [8.53] | 47 [9.53] | 366 [8.86] | 35 [8.91] | 12 [11.88] | 72 [13.04] |
| >34 | 4877 [4.30] | 997 [7.38] | 243 [6.31] | 754 [7.80] | 697 [7.80] | 275 [6.80] | 35 [7.10] | 332 [8.03] | 30 [7.63] | 18 [17.82] | 47 [8.51] |
| unknown | 40,188 [35.47] | 3965 [29.34] | 1150 [29.87] | 2815 [29.12] | 2622 [29.32] | 1291 [31.92] | 156 [31.64] | 1219 [29.50] | 101 [25.70] | 18 [17.82] | 154 [27.90] |
| BMI, mean [SD] | 24.83 [5.80] | 25.99 [6.54] | 25.55 [6.37] | 26.16 [6.59] | 26.19 [6.59] | 25.94 [6.41] | 26.36 [6.36] | 26.11 [6.53] | 25.74 [6.74] | 28.39 [7.95] | 26.73 [6.73] |

Exclusions: congenital anomalies and TOPFAs

Abbreviations as in Table 1.

Upward trends in asthma prescribing parallel those reported in England for people aged 15-44 2001-2005 [Simpson et al 2010].
