# Supplementary material for: Medicines prescribed for asthma, discontinuation and perinatal outcomes, including breastfeeding: A population cohort analysis
Source: PLoS One. 2020 Dec 9;15(12):e0242489. doi: 10.1371/journal.pone.0242489 (PMC7725302; doi:10.1371/journal.pone.0242489)
Supplement: S2 Table — (DOCX) [file pone.0242489.s003.docx]

Supplementary material

#### S2 Table Demographic details of subjects included in the analysis [n = 107573]

|  | **Population [exclusions listed below]** | **Any asthma medicine [R03 PRE4-T3]** | **Unmedicated asthma [R03 PRE4-1 and not T123]** | **Any asthma medicine t1-t3 [R03]** | **SABA t1-t3** | **Only SABA prescribed** | **LABA t1-t3**  **[R03AC12,13]** | **ICS t1-t3 [R03BA]** | **Only ICS prescribed** | **LKA t1-t3 [R03DC]** | **OCS t1-t3 + another asthma medicine**  **[H02AB + R03]** |
| --- | --- | --- | --- | --- | --- | --- | --- | --- | --- | --- | --- |
| **Number of infants** | n[%] | n[%] | n[%] | n[%] | n[%] | n[%] | n[%] | n[%] | n[%] | n[%] | n[%] |
| **Total** | 107,573 [100] | 12,690 [100] | 3589 [100] | 9101 [100] | 8413 [100] | 3820 [100] | 465 [100] | 3875 [100] | 374 [100] | 89 [100] | 519 [100] |
| **Parity:** |  |  |  |  |  |  |  |  |  |  |  |
| Primiparious | 45,483 [42.3] | 5380 [42.4] | 1605 [44.7] | 3775 [41.5] | 3499 [41.6] | 1594 [41.7] | 181 [38.9] | 1649 [42.6] | 160 [42.8] | 30 [33.7] | 159 [30.6] |
| Multiparious | 62,090 [57.7] | 7310 [57.6] | 1984 [55.3] | 5326 [58.5] | 4914 [58.4] | 2226 [58.3] | 284 [61.1] | 2226 [57.4] | 214 [57.2] | 59 [66.3] | 360 [69.4] |
|  |  |  |  |  |  |  |  |  |  |  |  |
| **Mean age** at pregnancy end [years: [SD]] | 28.20 [6.06] | 27.70 [6.1] | 27.14 [6.11] | 27.93 [6.1] | 27.84 [6.1] | 27.85 [6.1] | 28.47 [6] | 28.41 [5.86] | 28.43 [6.2] | 28.90 [5.88] | 28.64 [6.3] |
| <20 | 9114 [8.5] | 1265 [10.0] | 416 [11.6] | 849 [9.3] | 793 [9.4] | 355 [9.3] | 29 [6.2] | 387 [10.0] | 34 [9.1] | 6 [6.7] | 43 [8.3] |
| 20-24 | 22,808 [21.2] | 2914 [23] | 905 [25.2] | 2009 [22.1] | 1890 [22.5] | 865 [22.6] | 99 [21.3] | 876 [22.6] | 74 [19.8] | 12 [13.5] | 97 [18.7] |
| 25-29 | 29,415 [27.3] | 3551 [28] | 996 [27.8] | 2555 [28.1] | 2368 [28.1] | 1081 [28.3] | 148 [31.8] | 1061 [27.4] | 107 [28.6] | 30 [33.7] | 144 [27.7] |
| 30-34 | 28,716 [26.7] | 3055 [24.1] | 796 [22.2] | 2259 [24.8] | 2067 [24.6] | 929 [24.3] | 105 [22.6] | 946 [24.4] | 94 [25.1] | 24 [27] | 133 [25.6] |
| 35-39 | 14,577 [13.6] | 1576 [12.4] | 403 [11.2] | 1173 [12.9] | 1068 [12.7] | 487 [12.7] | 64 [13.8] | 505 [13.0] | 55 [14.7] | 11 [12.4] | 80 [15.4] |
| 40-44 | 2824 [2.6] | 314 [2.5] | 73 [2] | 241 [2.6] | 213 [2.5] | 98 [2.6] | 20 [4.3] | 99 [2.6] | 10 [2.7] | 6 [6.7] | 22 [4.3] |
| >44 | 111 [0.1] | 14 [10] | 0 | 14 [0.1] | 13 [0.1] | 5 [0.1] | § | § | § | § | § |
| unknown | 8 [0.0] | 1 [0.0] | 0 | 1 [0] | 1 [0] | 0 | 0 | 1 [0.0] | 0 | 0 | 0 |
| **Smoking status** |  |  |  |  |  |  |  |  |  |  |  |
| non-smoker | 60,901 [56.6] | 6661 [52.5] | 1857 [51.7] | 4804 [52.8 | 4395 [52.2] | 1948 [51] | 255 [54.8] | 2051 [52.9] | 232 [62] | 59 [66.3] | 252 [48.6] |
| current smoker | 31,069 [28.9] | 4165 [32.8] | 1218 [33.9] | 2947 [32.4] | 2776 [33] | 1314 [34.4] | 135 [29] | 1254 [32.4] | 93 [24.9] | 20 [22.5] | 185 [35.6] |
| ex-smoker | 13,428 [12.5] | 1756 [13.8] | 476 [13.3] | 1280 [14.1] | 1178 [14] | 520 [13.6] | 70 [15.1] | 544 [14.0] | 46 [12.3] | 10 [11.2] | 81 [15.6] |
| unknown | 2175 [2.0] | 108 [0.8] | 38 [1.1] | 70 [0.7] | 64 [0.7] | 38 [1] | 5 [1.1] | 26 [0.7] | 3 [0.8] | 0 | 1 [0.2] |
| **Socioeconomic status** |  |  |  |  |  |  |  |  |  |  |  |
| Wales Townsend fifth = 1 [least deprived] | 18,910 [17.6] | 2022 [15.9] | 549 [15.3] | 1473 [16.2] | 1328 [15.8] | 636 [16.6] | 78 [16.8] | 630 [16.3] | 83 [22.2] | 13 [14.6] | 70 [13.5] |
| 2 | 20,674 [19.2] | 2260 [17.8] | 703[19.6] | 1557 [17.1] | 1431 [17] | 674 [17.6] | 81 [17.4] | 627 [16.2] | 66 [17.6] | 12 [13.5] | 90 [17.3] |
| 3 | 20,961 [19.5] | 2476 [19.5] | 728 [20.3] | 1748 [19.2] | 1612 [19.2] | 765 [20] | 72 [15.5] | 700 [18.1] | 70 [18.7] | 16 [18] | 99 [19.1] |
| 4 | 22,934 [21.3] | 2789 [22] | 730 [20.3] | 2059 [22.6] | 1910 [22.7] | 823 [21.5] | 102 [21.9] | 891 [23.0] | 79 [21.1] | 26 [29.2] | 127 [24.5] |
| 5 [most deprived] | 23,662 [22.0] | 3095 [24.4] | 856 [23.9] | 2239 [24.6] | 2109 [25.1] | 913 [23.9] | 131 [28.2] | 1012 [26.1] | 74 [19.8] | 22 [24.7] | 131 [25.2] |
| Unknown | 432 [0.4] | 48 [0.4] | 23 [0.6] | 25 [0.3] | 23 [0.3] | 9 [0.2] | 1 [0.2] | 15 [0.4] | 2 [0.5] | 0 | 2 [0.4] |
| Townsend score, mean [SD] | 0.28 [3.17] | 0.51 [3.1] | 0.47 [3.1] | 0.52 [3.1] | 0.57 [3.19] | 0.46 [3.1] | 0.75 [3.4] | 0.49 [3.19] | -0.01 [3.0] | 0.52 [2.7] | 0.66 [3.1] |
| Townsend fifth, mean [SD] | 3.11 [1.41] | 3.21 [1.4] | 3.18 [1.3] | 3.22 [1.4] | 3.24 [1.4] | 3.18 [1.4] | 3.27 [1.4] | 3.22 [1.43] | 2.99 [1.4] | 3.36 [1.37] | 3.31 [1.3] |
| **Mean time on database at LMP [years]** | 7.62 [4.94] | 8.09 [5.05] | 8.07 [4.9] | 8.10 [5.08] | 8.14 [5.1] | 7.90 [4.7] | 7.36 [4.9] | 8.01 [5.03] | 7.14 [4.5] | 9.62 [5.74] | 8.02 [5.2] |
| **Pregnancy end date in** |  |  |  |  |  |  |  |  |  |  |  |
| 2000 | 7642 [7.1] | 742 [5.8] | 194 [5.4] | 548 [6] | 491 [5.8] | 269 [7] | 29 [6.2] | 257 [6.6] | 42 [11.2] | ˂5 | 26 [5] |
| 2001 | 7915 [7.4] | 814 [6.4] | 221 [6.2] | 593 [6.5] | 538 [6.4] | 266 [7] | 43 [9.2] | 295 [7.6] | 38 [10.2] | ˂5 | 19 [3.7] |
| 2002 | 8174 [7.6] | 857 [6.8] | 246 [6.9] | 611 [6.7] | 552 [6.6] | 255 [6.7] | 40 [8.6] | 318 []8.2 | 40 [10.7] | ˂5 | 24 [4.6] |
| 2003 | 9109 [8.5] | 1048 [8.3] | 280 [7.8] | 768 [8.4] | 711 [8.5] | 325 [8.5] | 61 [13.1] | 369 [9.5] | 34 [9.1] | ˂5 | 32 [6.2] |
| 2004 | 9766 [9.1] | 1146 [9] | 328 [9.1] | 818 [9] | 751 [8.9] | 337 [8.8] | 52 [11.2] | 385 [9.9] | 33 [8.8] | 6 [6.7] | 46 [8.9] |
| 2005 | 10,132 [9.4] | 1209 [9.5] | 318 [8.9] | 891 [9.8] | 836 [9.9] | 381 [10] | 47 [10.1] | 394 [10.2] | 32 [8.6] | ˂5 | 45 [8.7] |
| 2006 | 10,487 [9.7] | 1313 [10.3] | 371 [10.3] | 942 [10.4] | 864 [10.3] | 389 [10.2] | 59 [12.7] | 392 [10.1] | 40 [10.7] | 12 [13.5] | 58 [11.2] |
| 2007 | 10,949 [10.2] | 1379 [10.9] | 394 [11] | 985 [10.8] | 916 [10.9] | 411 [10.8] | 42 [9] | 392 [10.1] | 31 [8.3] | 6 [6.7] | 64 [12.3] |
| 2008 | 11,074 [10.3] | 1383 [10.9] | 438 [12.2] | 945 [10.4] | 892 [10.6] | 380 [9.9] | 33 [7.1] | 360 [9.3] | 23 [6.1] | 18 [20.2] | 61 [11.8] |
| 2009 | 10,948 [10.2] | 1319 [10.4] | 365 [10.2] | 954 [10.5] | 894 [10.6] | 398 [10.4] | 27 [5.8] | 325 [8.4] | 25 [6.7] | 15 [16.9] | 67 [12.9] |
| 2010 | 11,377 [10.6] | 1480 [11.7] | 434 [12.1] | 1046 [11.5] | 968 [11.5] | 409 [10.7] | 32 [6.9] | 388 [10.0] | 36 [9.6] | 19 [21.3] | 77 [14.8] |
| **Body mass index [BMI] nearest before LMP** |  |  |  |  |  |  |  |  |  |  |  |
| <20 | 8677 [8.1] | 906 [7.1] | 286 [8] | 620 [6.8] | 565 [6.7] | 243 [6.4] | 32 [6.9 | 282 [7.3] | 32 [8.6] | 10 [11.2] | 42 [8.1] |
| 20 – 24 | 31,869 [29.6] | 3646 [28.7] | 1053 [29.3] | 2593 [28.5] | 2374 [28.2] | 1091 [28.6] | 119 [25.6] | 1084 [28.0] | 120 [32.1] | 17 [19.1] | 122 [23.5] |
| 25 – 29 | 16,923 [15.7] | 2332 [18.4] | 654 [18.2] | 1678 [18.4] | 1569 [18.6] | 684 [17.9] | 91 [19.6] | 710 [18.3] | 63 [16.8] | 21 [23.6] | 97 [18.7] |
| 30 – 34 | 7178 [6.7] | 1140 [9] | 296 [8.2] | 844 [9.3] | 779 [9.3] | 321 [8.4] | 43 [9.2] | 340 [8.8] | 34 [9.1] | 11 [12.4] | 65 [12.5] |
| >34 | 4609 [4.3] | 936 [7.4] | 224 [6.2] | 712 [7.8] | 656 [7.8] | 258 [6.8] | 34 [7.3] | 317 [8.2] | 29 [7.8] | 15 [16.9] | 42 [8.1] |
| unknown | 38,317 [35.6] | 3730 [29.4] | 1076 [30] | 2654 [29.2] | 2470 [29.4] | 1223 [32] | 146 [31.4] | 1142 [29.5] | 96 [25.7] | 15 [16.9] | 151 [29.1] |
| BMI, mean [SD] | 24.83 [5.79] | 25.98 [6.4] | 25.94 [6.2] | 26.15 [6.5] | 26.18 [6.5] | 25.91 [6.3] | 26.33 [6.3] | 26.10 [6.52] | 25.69 [6.5] | 28.12 [7.9] | 26.53 [6.6] |

Notes: Exclusions: all congenital anomalies, terminations of pregnancy for foetal anomalies [TOPFA], stillbirths, multiple births [twins, triplets and quadruplets [no higher multiples in the dataset]], exposure to insulin, anti-epileptic drugs [AEDs] or coumarins in the quarter preceding pregnancy and trimester 1, heavy drinking/substance misuse [any record].

Some numbers have been blurred to avoid revealing numbers <5 in other cells.

§ numbers in this category <5, and therefore combined with the category above to avoid disclosure of low numbers.

Abbreviations as in Table 1.

Upward trends in asthma prescribing parallel those reported in England for people aged 15-44 2001-2005 [Simpson et al 2010].
