# Supplementary material for: Medicines prescribed for asthma, discontinuation and perinatal outcomes, including breastfeeding: A population cohort analysis
Source: PLoS One. 2020 Dec 9;15(12):e0242489. doi: 10.1371/journal.pone.0242489 (PMC7725302; doi:10.1371/journal.pone.0242489)
Supplement: S3 Table — (DOCX) [file pone.0242489.s004.docx]

#### S3 Table Medicated and unmedicated asthma: descriptive data n= 107573

|  | **asthma [R03 in Pre1234 or T123]** | | **medicated [in T123] asthma [R03]** | | **unmedicated [in Pre1234 but not T123] asthma [R03]** | |
| --- | --- | --- | --- | --- | --- | --- |
|  | Exposed n [%] | Unexposed [n [%] | Exposed n [%] | Unexposed [n [%] | Exposed n [%] | Unexposed [n [%] |
| 2000-2010 |  |  |  |  |  |  |
| **Preterm birth** |  |  |  |  |  |  |
| ≥37 weeks' gestation | 11,907 [93.83] | 89,509 [94.34] | 8563 [94.09] | 92,853 [94.29] | 3344 [93.17] | 98,072 [94.31] |
| <37 to  32 weeks' gestation | 652 [5.14] | 4572 [4.82] | 450 [4.94] | 4774 [4.85] | 202 [5.63] | 5022 [4.83] |
| <32 weeks' gestation | 131 [1.03] | 802 [0.85] | 88 [0.97] | 845 [0.86] | 43 [1.2] | 890 [0.86] |
| total | 12,690 [100] | 94,883 [100] | 9101 [100] | 98,472 [100] | 3589 [100] | 103,984 [100] |
| all <37 weeks' combined | 783 [6.17] | 5374 [5.66] | 538 [5.91] | 5619 [5.71] | 245 [6.83] | 5912 [5.69] |
| **SGA** |  |  |  |  |  |  |
| ≥10th centile | 11,221 [88.42] | 84,953 [89.53] | 8059 [88.55] | 88,115 [89.48] | 3162 [88.1] | 93,012 [89.45] |
| <10th to 3rd centile | 932 [7.34] | 6258 [6.6] | 666 [7.32] | 6524 [6.63] | 266 [7.41] | 6924 [6.66] |
| <3rd centile | 260 [2.05] | 1707 [1.8] | 179 [1.97] | 1788 [1.82] | 81 [2.26] | 1886 [1.81] |
| unknown | 277 [2.18] | 1965 [2.07] | 197 [2.16] | 2045 [2.08] | 80 [2.23] | 2162 [2.08] |
| total | 12,690 [100] | 94,883 [100] | 9101 [100] | 98,472 [100] | 3589 [100] | 103,984 [100] |
| all <10th centile | 1192 [9.39] | 7965 [8.39] | 845 [9.28] | 8312 [8.44] | 347 [9.67] | 8810 [8.47] |
| 2004-2010 |  |  |  |  |  |  |
| **breastfeeding** |  |  |  |  |  |  |
| at birth |  |  |  |  |  |  |
| yes | 3064 [24.14] | 22,061 [23.25] | 2202 [24.2] | 22,923 [23.28] | 862 [24.02] | 24,263 [23.33] |
| no | 2967 [23.38] | 19,634 [20.69] | 2088 [22.94] | 20,513 [20.83] | 879 [24.49] | 21,722 [20.89] |
| unknown | 6659 [52.47] | 53,188 [56.06] | 4811 [52.86] | 55,036 [55.89] | 1848 [51.49] | 57,999 [55.78] |
| total | 12,690 [100] | 94,883 [100] | 9101 [100] | 98,472 [100] | 3589 [100] | 103,984 [100] |
| at 6-8 weeks |  |  |  |  |  |  |
| yes | 1499 [11.81] | 11,294 [11.9] | 1086 [11.93] | 11,707 [11.89] | 413 [11.51] | 12,380 [11.91] |
| no | 3416 [26.92] | 22,516 [23.73] | 2433 [26.73] | 23,499 [23.86] | 983 [27.39] | 24,949 [23.99] |
| unknown | 7775 [61.27] | 61,073 [64.37] | 5582 [61.33] | 63,266 [64.25] | 2193 [61.1] | 66,655 [64.1] |
| total | 12,690 [100] | 94,883 [100] | 9101 [100] | 98,472 [100] | 3589 [100] | 103,984 [100] |

Exclusions: all anomalies [including TOPFAs], stillbirths, multiples [non - singletons], exposed to insulin, AEDs or coumarins in the quarter preceding pregnancy or trimester 1, exposed to heavy drinking/substance misuse [any record]
