# Supplementary material for: Medicines prescribed for asthma, discontinuation and perinatal outcomes, including breastfeeding: A population cohort analysis
Source: PLoS One. 2020 Dec 9;15(12):e0242489. doi: 10.1371/journal.pone.0242489 (PMC7725302; doi:10.1371/journal.pone.0242489)
Supplement: Attachment — Submitted filename: PONE-D-20-02812 r2r.docx [file pone.0242489.s005.docx]

Changes Made

| Reviewer #1: |  |
| --- | --- |
| The study covers a useful research question and the data source is appropriate for answering the key questions. From my perspective, however, the study would require substantial changes to be useful for the intended audience. | Thank you. |
| These are my key observations: |  |
| 1) The objectives seem to be a mix of showing non causal associations and showing causal relationships. I think identifying associations is more appropriate without using more rigorous methods to account for confounding (e.g. propensity score methods). From this perspective, the current regression models would be what some people call “explanatory” models – i.e. to show various relationships – which is still useful. Some sections do, appropriately, focus on associations. | Thank you, we agree that causality cannot be determined from retrospective cohorts. We have clarified our aims and methods:  *To identify women likely to benefit from additional care, we... p.4*  *There was no randomisation, and, therefore, we explored association, rather than causality. P.4* |
| The methods, though, describe control for confounding which would imply the goal is causal inference – i.e. to show the extent to which these medications cause the adverse pregnancy outcomes described. I would expect that all of the exclusion criteria would tend to limit generalizability, but I don’t see that they address the key confounding issues. I also don’t see that the regression models get at the key problems. When, for example, you want to know the causal association between SABA use and preterm birth, the article raises the issue of controlling for “confounding by indication.” In this case, SABAs could be used in patients with more severe asthma or with poorly controlled asthma, and therefore it is difficult to distinguish adverse effects of the drug from those of the underlying disease.  To address confounding by indication you would need information on asthma severity (or other underlying causes of adverse outcomes) and use appropriate methods to address. The better alternative, from my perspective, is to simply treat this as descriptive data and not attempt to address confounding. You can still conduct multivariable modeling in the framework of a descriptive or explanatory regression model. You can also develop a “predictive” model but this would require a different strategy. | Thank you. We now offer a definition of confounding from a standard medical text, based on an earlier Lancet series. *Confounding is a blurring of effects when a variable is associated with the exposure and affects the outcome (Schulz & Grimes 2006 p.34). p.7*  *Restricting the dataset by excluding pregnancies exposed to AEDs, insulin, coumarins, heavy drinking or substance misuse and multiple pregnancies increases internal validity at the cost of generalisation to these groups (Schulz & Grimes 2006). P.22*  Information on severity of asthma was inferred from prescriptions. As referenced, in the UK, prescriptions follow the BTS and BNF guidelines (references [5, 28]). P.5 and Table 1. Prescribers adhere to these guidelines. For example, oral corticosteroids are only prescribed for severe asthma. |
|  |  |
| 2) I would have expected the study to be conducted among women with asthma. Including all pregnant women made the interpretation more difficult. | This is a population-based cohort. As healthcare databases become more available, such designs are increasingly common (Sterne et al 2016). We have added a sentence relating to their advantages p.4:  *A population-based cohort was built from prospectively collected routine NHS data and analysed retrospectively. Such cohorts have minimal selection and attrition bias and no recall bias (Thygesen and Ersboll 2014).* We explored association, rather than causality, as there was no randomisation. |
| 3) I found several sections of the manuscript difficult to follow. In some cases, it was because multiple ideas were combined in the same sentence. Here is an example: “Breastfeeding [any, even if supplemented by formula feeding] at birth and 6- 8 weeks is routinely recorded by health visitors, and this information is transferred to NCCHD; data collection is more complete in some Health Board regions than others. [33]  Breastfeeding at birth is considered an indirect measure, which may represent intention rather than practice, and is downgraded as evidence of successful breastfeeding. [34]” This section combined information on how data is collected (which I think fits better in the database description) and information on interpretation (does BF at birth correlate with later BF?), but doesn’t actually state how BF was defined for the analysis. I had trouble understanding what “indirect measure” meant (Indirect measure of longer term BF?) and what “is downgraded” meant (downgraded by other researchers?). And after all that, I still wasn’t clear on how BF was defined for the analysis, which was the main point of this section.  As another example, the phrase “% without unknown outcome” was confusing. | We have clarified, and added a reference p.6. *Breastfeeding in this database is defined as ‘any breastfeeding’ (exclusive, or combined, predominantly or partially, with other feeds, including formula milk) as recorded by health visitors and entered into NCCHD (NHS Wales 2017).*  We have reworded to clarify, thank you: *Breastfeeding at birth may represent intention to breastfeed, rather than actuality: a high proportion of dyads breastfeeding at birth have discontinued a week later. Therefore, this measure is downgraded as evidence of successful breastfeeding. [35]* p.7  % without ‘unknown outcome’ is analogous to ‘missing or outcome unknown’. This entire table has been removed, as it is now available as supplementary material in the paper using the same dataset, but exploring antidepressants (Jordan et al 2019). |
| 4) Starting the results and tables (e.g. Table 2) with an assessment of women excluded from your analysis was confusing. (Why focus the reader on these results if these women were not even eligible for the analysis?) I could see this assessment as a sensitivity analysis after presenting main results. | This table is available in supplementary material in association with the co-submitted paper from the same data set. Therefore, it will not be submitted with this paper.  We believe this table contains very important information on the risks associated with other prescriptions, and will be of interest to those planning research in the area. |
| 5) The results were not calculated and reported in a standard and useful way. If I look at the first 2 rows of Table 2, for example, a more useful way to present the results is:  a. Among those exposed to insulin, 213/688 (35.6%) had preterm delivery. (688 = 231+457)  b. Among those not exposed, 7863/112,628 (7.5%) had preterm delivery  c. The risk ratio (or relative risk) is then 4.48  d. The odds ratio (6.73) is technically correct but it is more difficult to interpret and could be easily interpreted as overstating the strength of the relationship. The regression models can match by using a model that reports a risk ratio. Modified Poisson is one that is easy to conduct: <https://www.ncbi.nlm.nih.gov/pubmed/15033648> Log binomial can work as well although sometimes has convergence problems. | This table has been removed from the paper. Readers are referred to its publication elsewhere.  We agree that odds and risk ratios can be used to calculate these differences, and readers are provided with sufficient information to make their own calculations. The numbers of subjects in table 2 are, in our opinions, too low for multivariate analyses. |
| The tables report both the OR and the inverse of the OR which is confusing. In this case, the OR of 6.73 is the useful one for showing the relationship with increased risk of prematurity. The second OR (0.15) reversing the referent group is not useful. The tables should keep the referent group constant and identify the referent group (e.g. label cell as “ref”) and/or show an OR (or RR) of 1.0 for the referent group. The tables should also clearly delineate each association assessed. For example, the first 2 rows are one comparison (association between insulin and birth <37 weeks) and it would be helpful to distinguish that from subsequent comparisons. | This table has been removed from the paper. Readers are referred to its publication elsewhere.  The additional data indicate to readers that data are completely reported. |
| 6) For each association, the comparison group should be stated clearly. In the abstract, for example, when you state that discontinuation is associated with preterm delivery, what is this in comparison to? (to those who continued all their treatments? To all other women including those without asthma or those who weren’t treated at all?) | We are sorry if this is unclear. We state, p.8: *For each analysis we compared the exposed with the unexposed population.*  We have now added to the notes to table 2 and to the abstract: *Odds ratios for adverse pregnancy outcomes were calculated for the exposed versus the unexposed population.* |
| 7) The data set seems quite old. Treatment guidelines and treatment options (such as biologics) have changed since this time. More recent data would be more relevant, if available. | We agree. Were funding available, we would extend the study. However, this is the first report of the predictors of infant feeding and one of the most detailed reports available on medicine discontinuation in the ambulant population. We have used our data to identify how care can be improved, and hope that readers will consider this an important practice point.  We have added to the ‘limitations’ p.22: *Our data predate the primary care prescription of monoclonal antibodies for severe eosinophilic asthma persisting despite oral corticosteroid therapy. Manufacturers advise avoiding these medicines in pregnancy [28]; therefore, population databases may be unable to describe their safety in pregnancy for some time.* |
| Reviewer #2: |  |
| The authors have found an association of adverse perinatal outcomes in primary care setting, in Brittan, Wales. It is an area with the greatest prevalence of asthma in Europe. As asthma is the most common chronic disease among pregnant women, we should be very careful on drawing conclusions. As the asthma is a disease of many different phenotypes, severity and treatments, there is still need for more investigation such as this article. | Thank you. We agree, we cannot draw conclusions regarding aetiology, but we hope to suggest practice modifications. |
| The authors have shown for the first time that the discontinuation of the medication of active asthma during pregnancy increases risk of preterm birth. There have been articles previously showing that unmedicated asthma has similar effects. The oral glucocorticoids are not analyzed in many of the articles evaluating asthma. The risks in the different articles are not seen similar in relation to oral glucocorticoids. The authors have shown for the first time that maternal asthma carries a risk of lower breast feeding rate for the first time. | Thank you. |
| The authors present the literature in relation to asthma very well and their findings are in accordance to the previous literature. | Thank you. |
| 1) The terms hypoxia and asphyxia and their use should be controlled. As asthma may cause maternal hypoxia, the authors should be clear whether they mean maternal, placental, fetal, perinatal or neonatal hypoxia or asphyxia. This should be clear for the reader all times. | Thank you, we have corrected. We have retained ‘asphyxia’ just once, on p.20, line 344, where complications of SGA are quoted: *… range of conditions from neonatal asphyxia to mental health conditions in adulthood* |
| 2) The oral glucocorticoids are used for autoimmune diseases such as rheumatoid arthritis or inflammatory bowel diseases. These are associated with problems in pregnancies. Often people also have autoimmune tendency and suffer from several autoimmune diseases (asthma + RA+ IBD). It is also common for some patients to use short acting betasympathomimetics in pulmonary symptoms other than asthma. The authors have found association of worse prognosis associated with the oral glucocorticoid use. The authors have tried to make sure the use of oral corticosteroids is for asthma by necessitating the use of some other asthma medication. Have the authors information about the prevalence of the most common autoimmune diseases? If not, the authors should somehow comment the risk of confounding. | We agree, this is an important point. Only 5 women with type 1 diabetes were prescribed oral corticosteroids (S1 file, table A1). Only 519 pregnancies were exposed to oral corticosteroids. Although we excluded the commonest co-morbidities, where pregnancy risks are known to be higher than for asthma, we acknowledge that other, rarer, co-morbidity was not excluded, and have added to our ‘limitations’ section:  *Rarer co-morbidities were not excluded. Co-morbid rheumatoid arthritis and inflammatory bowel disease were more likely amongst women prescribed oral corticosteroids, who would have received specialist care [5}.*  We agree that beta2 adrenoceptor agonists are indicated for all conditions associated with reversible airways obstruction. In this age-group, asthma is the most common. We have added to notes to Table 1:  *SABA and LABA are indicated for all conditions associated with reversible airways obstruction [28].*  And to our discussion, final paragraph:  *Prescription patterns, regardless of diagnoses, particularly discontinuation in pregnancy, offer primary care nurses, midwives and nurse specialists a convenient marker to identify pregnancies at increased risk, analogous to an ‘intention to treat’ analysis: pragmatic but of unproven biological certainty.* |
| 4) Authors state in introduction without a reference that asthma medications cross placenta and enter breastmilk. Is this true for all the asthma medications and could the sentence just be left out? As the article is not about congenital anomalies, is it necessary to add articles about them in relation to asthma? | We apologise, and have added references. We suggest this short sentence supports the biological plausibility of our findings.  We have included the reference to congenital anomalies to alert future systematic reviewers, that this data set has been used for this outcome. It is important that future reviewers avoid all possible double-counting of events. |
| 5) Did the authors have information on just prescription or also on purchases and dispension? Please state clearly. | We apologise if this is not clear. We have clarified:  *We have no data on dispensing or hospital or private prescribing or internet purchasing. It is unlikely that anyone hospitalized with asthma or consulting privately would not be receiving prescriptions from primary care practitioners. Abolition of prescription charges in Wales in 2007 reduces the likelihood of private prescribing and purchasing.* |
| 6) This setting is primary care setting. The amounts of mediccations used differ a bit from the other studies. The secondary and tertiary care prescribtions on asthma medications are not included? Does this explain absence of some medication groups? Most of the asthma is managed in primary care, which make primary care settings more useful. | We hope the sentence above clarifies.  We agree, that our important findings relate to primary care, and the ‘less ill’ women. We think that even a 1% increase in preterm birth rate in these women is important, and might be avoided. |
